# Mathematical modeling and analysis for tissue curvature correction in near-infrared spectroscopy imaging

**DOI:** 10.1117/1.JBO.30.9.096002

**Published:** 2025-09-19

**Authors:** Himaddri Shakhar Roy, Daniela Leizaola, Charles Policard, Anuradha Godavarty

**Affiliations:** Florida International University, Optical Imaging Laboratory, Department of Biomedical Engineering, Miami, Florida, United States

**Keywords:** near-infrared spectroscopy, tissue oxygenation, diabetic foot ulcers, smartphone-based imaging device, tissue curvature, curvature correction, Monte Carlo light propagation model, optical phantoms, diffuse reflectance, hemoglobin parameters

## Abstract

**Significance:**

Near-infrared spectroscopy (NIRS) imaging modalities are used to provide noncontact measurements of tissue oxygenation in diabetic foot ulcers. However, the curved surface of the diabetic foot introduces inaccurate tissue oxygenation measurement. The changes in spatial NIRS optical measurements may result from variations in the underlying physiology or from the curvature of the tissue surface. Therefore, the effect of tissue curvature must be accounted for to ensure the accurate measurement of tissue oxygenation (or hemoglobin parameters) in clinical applications.

**Aim:**

Our aim is to develop and validate mathematical curvature correction models to account for the effects of tissue curvature on diffuse reflectance (DR) in NIRS imaging and assess their effect on the hemoglobin parameters as well.

**Approach:**

Monte-Carlo-based light propagation simulations were performed to develop correction models and applied to three-layered curved geometries in MCMatlab. Four curvature correction models based on height and/or angle were developed via Monte Carlo simulation studies. All the correction models were applied to the simulated DR signals obtained from various curved geometries (concave, convex, and wound-mimicking) using Gaussian light sources at 690 and 830 nm. The effect of correction models on DR signals and hemoglobin parameters was determined.

**Results:**

Simulation results showed that a concave curved surface did not require correction, whereas convex and wound-mimicking geometries showed a reduced median error upon using an empirical height/angle correction model. In addition, the correction model also reduced the median error significantly for the oxygen-saturation-based hemoglobin parameter in both the convex and wound-mimicking geometries.

**Conclusions:**

The developed mathematical model effectively corrected tissue curvature effects in NIRS DR signals and hemoglobin parameters for wound-mimicking irregular geometry. Ongoing work focuses on experimental validation of these correction models on curved phantoms, prior to *in vivo* imaging studies.

## 1 Introduction

Diabetic foot ulcers (DFUs) cause serious complications of diabetes mellitus (DM), contributing to major health challenges due to infection, amputation, or death.^1^ It is estimated that 689 million people worldwide will have diabetes mellitus by 2024, and the number might rise to 853 million by 2050.^2^ One in three people with diabetes mellitus are affected by DFUs,^3^ and 20% of them result in lower limb amputations.^4^ Therefore, effective management of DFUs is essential to improve the quality of life of DM patients.^5^ Unlike the gold-standard clinical visual assessment of the wound size and granulation features to assess healing potential, objective optical imaging approaches [e.g., hyperspectral imaging (HSI), multispectral imaging (MSI), and near-infrared spectroscopy (NIRS)] have been developed^6^^,^^7^ to map tissue oxygenation changes in and around the wounds to physiologically assess wound healing potential for improved wound care management.

In the past, we developed a smartphone-based NIRS imaging device (smartphone-based oxygenation tool, SPOT) that is capable of noncontact wide-field imaging for tissue oxygenation changes. The NIRS-based SPOT device has been used to image and assess DFUs in our past studies.^8^^–^^11^

Noncontact-based optical imaging (NIRS, HSI, or MSI) of tissue regions is impacted by tissue curvatures. This is because the area-based detectors (e.g., CCD or CMOS cameras) capture the diffuse reflectance (DR) signals from a focused detection plane that is flat, although the tissue regions are typically curved (and not flat). The signal loss due to differences in the depth between the flat detection plane and the tissue curvature, as well as the angle of illumination and detection with respect to the curved tissue surface, can affect the DR signals apart from the underlying physiology that alters the DR signals. The foot in general has an irregular tissue geometry that is not flat, and when imaged for tissue oxygenation around the DFUs, it can give errors due to light losses from tissue curvature effects. Hence, the effects of tissue curvature should be corrected to obtain the true tissue oxygenation changes due to the underlying physiology and not due to curvature effects. The objective of this study is to develop and implement mathematical correction techniques on DR signals from curved geometries via extensive Monte Carlo (MC) simulation studies and analyze the effect of tissue curvature on tissue oxygenation parameters as well.

Several techniques have been reported in the literature for correcting curvature effects. The techniques include measuring the 3D height and angular distribution of the object and applying the correction. The summary of all the past work is given in Table 1. The table describes the correction models applied to the various optical imaging techniques and parameters corrected via experimental studies.

**Table 1:** Summary of key studies addressing light illumination, imaging techniques, and correction methods for accurate depth extraction and application in different imaging systems.

Refs.	Light illumination	Imaging techniques	Height correction	Angle correction	Experimental studies	Device for depth extraction	Parameter corrected
Gioux et al.^12^	Quartz tungsten halogen lamp	Modulated imaging	Yes	Yes	Hemispheric phantom and human fingers	Phase profilometry	Absorption and reduced scattering
Kainerstorfer et al.^13^	Uniform illumination (halogen white light source)	MSI	No	Yes	Cylindrical phantom and *in vivo* (lower arm)	None used	Fraction blood oxygenation and fraction blood volume
Zhao et al.^14^	LED illumination (did not mention about distribution)	SFDI	Yes	Yes	Semi-hemisphere phantoms and *in vivo* (rat wound)	Optical profilometry	Absorption, reduced scattering, and total hemoglobin
Rogelj et al.^15^	LED illumination (homogeneous illumination)	HSI	Yes	Yes	Hemisphere, toy figure, and a human finger	3D profilometry	Absorption concentration, melanin, deoxyhemoglobin, and oxyhemoglobin

In all these past studies, the assumption was that the light source has uniform illumination.^12^^,^^13^^,^^15^ The studies reported methodologies that implemented only the angle correction^13^ or incorporated both height and angle correction to improve the accuracy of the measured signal.^12^^,^^14^^,^^15^ These corrections have been implemented in various optical imaging techniques, including MSI,^13^ spatial frequency domain imaging (SFDI),^12^^,^^14^ and HSI.^15^ The corrections have been applied and validated via experimental studies on hemispherical and cylindrical tissue-mimicking phantoms and *in vivo* studies. In addition, phase profilometry^12^ or optical profilometry^14^^,^^15^ has been used to determine the surface depth and angle when implementing the correction factors experimentally on the imaged tissues or tissue-mimicking phantoms.

Although the height and angle correction models have been developed for uniform light illumination, they are not applicable when the light is not uniformly illuminated. In our custom-developed smartphone-based NIRS optical device (SPOT), the light distribution is not uniform but close to a Gaussian distribution. Hence, in this study, we developed appropriate curvature correction models for our current NIRS imaging device and tested them via extensive simulated studies on phantoms with varying curvature geometries.

## 2 Methodology

Curvature correction includes the correction of DR signals of the three-layer curved tissue phantom of varying curved geometries. A three-layered flat phantom, including the epidermis, dermis, and subcutis layer model, was developed in MCMatlab^16^ with tissue-mimicking optical properties (Table 2) and simulated with 1 billion photons. The thickness^17^ and optical properties, i.e., absorption coefficient^18^ ($\mu_{a}$), scattering coefficient^18^ ($\mu_{a}$), anisotropy factor^19^ (g), and refractive index^20^^,^^21^ (n) of epidermis, dermis, and subcutis layer were obtained from literature. The optical properties of the epidermis layer were empirically chosen to mimic Fitzpatrick skin type 2. The optical properties of the calibration sheet were simulated (within the range of a typical tissue, as given in Table 2) and used as a reference signal to calculate the hemoglobin parameters (as described in detail in Sec. 3.2).

**Table 2:** Optical and geometric parameters used in Monte-Carlo simulations with MCMatlab for multilayer skin modeling.

Simulation parameters		Values							
Number of photons		1 billion							
Model dimension		$10\times 10\times 6\;{\mathrm{cm}}^{3}$							
Light source		Uniform and Gaussian							
Simulation wavelengths		690 and 830 nm							
Tissue optical properties									
Layer	Thickness (cm)	690 nm				830 nm			
		$\mu_{a}$ (${\mathrm{cm}}^{-1}$)	$\mu_{s}$ (${\mathrm{cm}}^{-1}$)	g	n	$\mu_{a}$ (${\mathrm{cm}}^{-1}$)	$\mu_{s}$ (${\mathrm{cm}}^{-1}$)	g	n
Epidermis	0.0206	4.9403	161.1201	0.8201	1.431	2.6727	172.9819	0.8607	1.431
Dermis	0.30	0.37159	105.1904	0.8201	1.378	0.42059	94.5299	0.8607	1.378
Subcutis	3.6794	1.15	147.3016	0.96	1.44	1.05	131.5236	0.96	1.44
Calibration sheet	0.2	0.20	200	0.90	1.40	0.10	150	0.90	1.40

A uniform light source was used while developing correction factors to avoid any angle discrepancy between the surface normal and the illumination direction (as described in Sec. 2.1). Height and angle-based correction models were developed via MCMatlab simulation studies and further used to correct DR signals for different curved surfaces.

A Gaussian light source was used to illuminate the curved phantoms while implementing the curvature correction factors (as described in Sec. 2.2). A focused Gaussian light source was modeled using a combination of spatial and angular Gaussian distributions. The intensity distribution in the focal plane was defined as a symmetric two-dimensional Gaussian profile with 1.5 cm in both the X and Y directions to mimic the Gaussian light source from our SPOT device. Although the distance between the source and the detection plane was reduced from 4.5 cm (typically used in our experimental studies) to 2 cm (in our current simulation studies), the area of illumination was maintained the same (∼3  cm radius) as that obtained using our SPOT device during experimental studies. Although SPOT acquires images from 10  cm×6  cm area during experimental studies (optimized based on signal-to-noise calculations in our past studies^22^), herein the detected area was kept the same as the entire top surface of the phantom (10  cm×10  cm area) during simulation studies.

These simulated Gaussian light sources at 690 and 830 nm wavelengths were used to illuminate the simulated phantoms and obtain the DR signals from the top surface of the phantoms.

The effect of the developed correction models on DR signals (at both wavelengths) and the derived hemoglobin parameters was determined (described in detail in Sec. 3.2). Details of the developed correction models and their effect when implemented on various curved geometries are described in detail in the following sections.

### 2.1 Correction Models

The correction models include (1) height correction (ϵ),^12^^,^^15^^,^^23^ (2) Lambertian angle correction (cos θ),^12^ (3) both height and angle correction from (1) and (2), and (4) empirical correction.^24^

#### 2.1.1 Model 1: height correction

A height correction model has been developed to compensate for the difference in height from the imaged phantom surface to the focused imaging plane of the detector. Height correction can be developed using experimental calibration or the inverse square law model.^12^

**Fig. 1:**
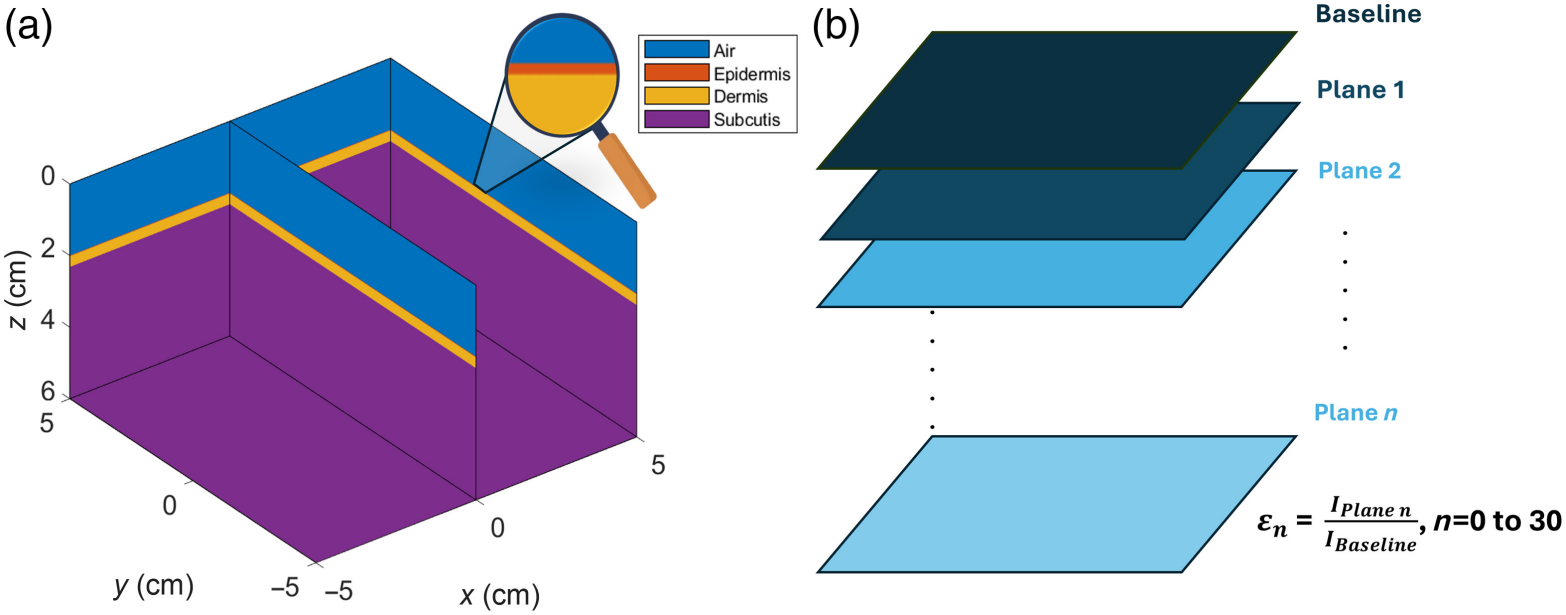
(a) Three-layered flat tissue phantom model and (b) method for calculating height correction factor ($\varepsilon_{n}$) across varying phantom elevations. The phantom consists of three biological tissue-mimicking layers: epidermis (red), dermis (yellow), and subcutis (purple), topped with an air interface (blue). The signal was collected from the air layer above the epidermis layer at a fixed position, perpendicular to the z-axis (baseline). (b) The phantom height is varied by incrementally varying the entire model along the z-axis in a step size of 0.1 cm, forming a series of varying planes n (n=0 to 30). Here, plane 0 corresponds to the baseline. The height correction factor, $\varepsilon_{n}$, was developed as a function of the distance between the flat phantom surface and the focused imaging plane (n).

**Fig. 2:**
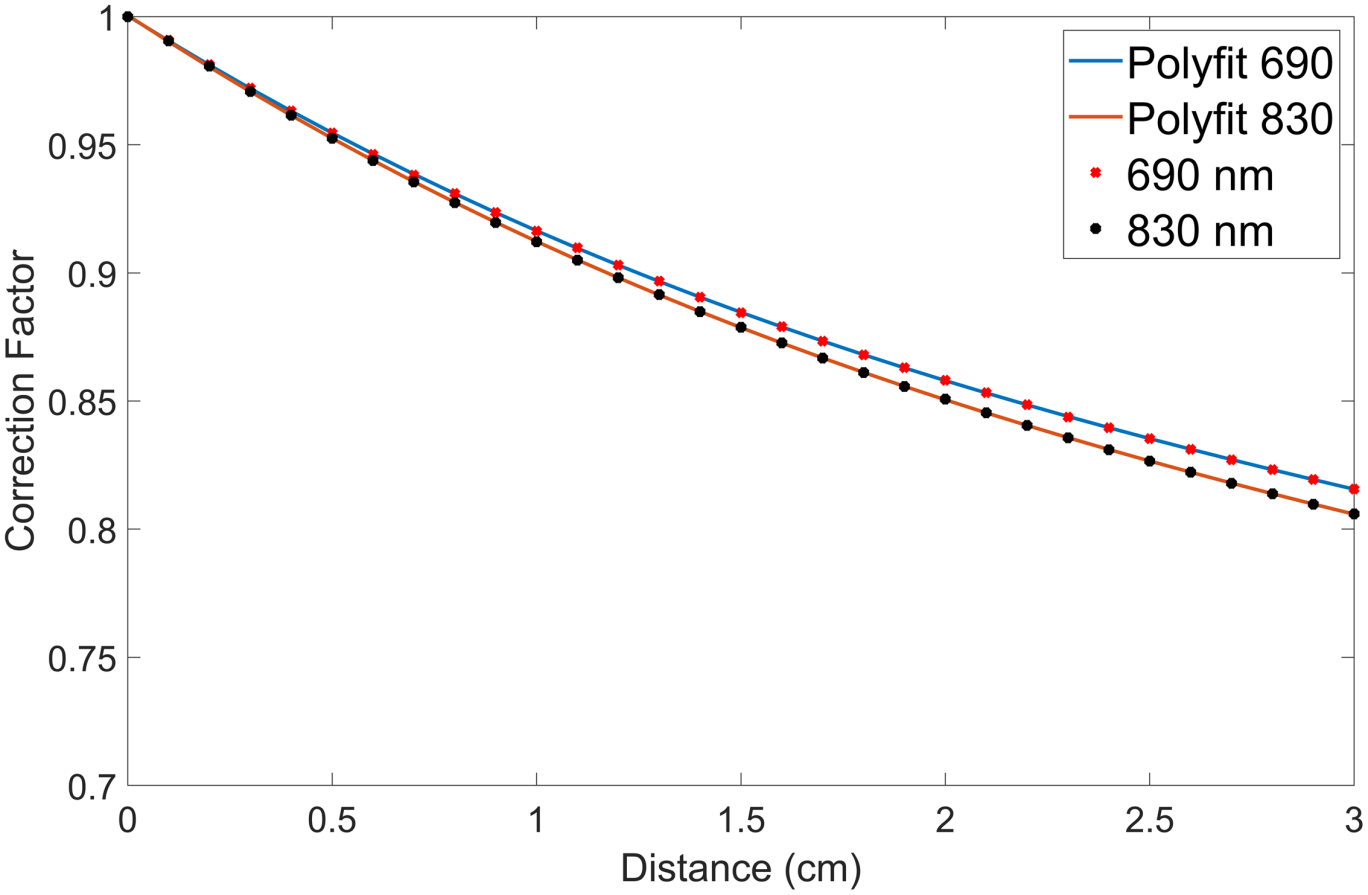
Height correction factor (ε) versus phantom elevation distance for two wavelengths: 690 (blue solid line) and 830 nm (red solid line). The x-axis represents the vertical displacement of the phantom surface from the baseline position (Δz), and the y-axis represents the height correction factor. The dots represent the raw correction factor values obtained from simulation measurements at each wavelength, and the solid lines represent polynomial fits of the correction factors.

The experimental calibration method was used for height correction due to its easier adaptability to various geometries.^15^ The correction model has been developed with respect to the flat phantom surface and later applied to different curved surfaces during the testing phase (see Fig. 1). A simulated flat three-layered phantom [Fig. 1(a)] has been used during the height correction model development. The DR signals were collected from a fixed distance from the top of the flat phantom surface via MC simulation studies. The distance between the detector (camera) and imaging plane was kept constant, and the distance between the focused imaging plane and the flat phantom surface (Δz) varied from 0 to 3 cm at 0.1 cm increments (i.e., a total of n=30 planes) to collect DR signals. The height correction factor ε was estimated as the ratio of the mean baseline DR signal (when the flat phantom surface and the focused imaging plane matched) to the mean DR signal obtained across the incremental distances between the flat phantom surface and the focused imaging plane [Fig. 1(b)]. A quadratic function of the height correction factor, ε, was developed as a function of the distance between the flat phantom surface and the focused imaging plane (Δz) (see Fig. 2). This height correction factor was developed at both the NIR wavelengths (690 and 830 nm). The quadratic function developed (with $R^{2}=1.0$ at both 690 and 830 nm) was further used when correcting for the height difference between the focused imaging plane and the curved phantom surface at each location (x,y) as given by

$$I_{H\_\mathrm{Corr}}(x,y)=\frac{I_{\text{raw}}(x,y,\Delta z)}{\varepsilon (\Delta z)},$$ (1)

where $I_{H\_\mathrm{Corr}}$ is the height-corrected DR signal, $I_{\text{raw}}$ is the raw DR signal, and ε is the height correction factor.

#### 2.1.2 Model 2: Lambertian angle correction

**Fig. 3:**
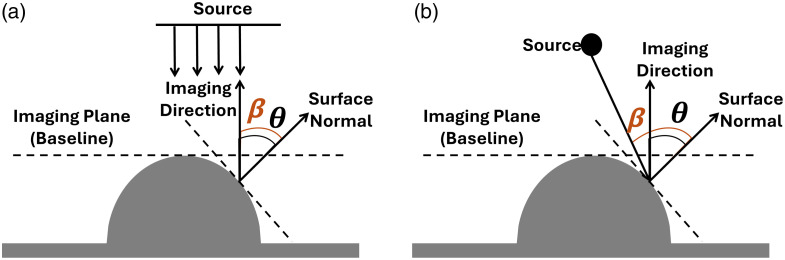
Schematic representation of angle-dependent signal collection from a curved tissue surface for (a) uniform light source and (b) Gaussian light source, and the requirement of correction of curved surfaces. As the detector collects tangential signals, surface curvature introduces an inclination angle (θ) between the surface normal and the imaging plane (baseline).

A Lambertian angle correction model was implemented as our second model. The intensity of the detected DR signal is directly proportional to the angle between the imaging direction (or detection direction, which is perpendicular to the imaging plane) and the surface normal. To eliminate the effect of curvature, this DR signal should be corrected with respect to the angle between the imaging direction and the surface normal. Hence, the angle correction factor (cos θ) was determined based on the angle between the tangent of the detection plane at each pixel and the surface normal of the curved geometry (see Fig. 3). The emitted DR signal from the surface element to the detector is reduced by the cosine of the emitted angle.^15^ The correction of the detected DR signal ($I_{A\_\mathrm{Corr}}$) can be applied as

$$I_{A\_\mathrm{Corr}}(x,y)=\frac{I_{\text{raw}}(x,y)}{\cos\;\theta },$$ (2)

where cos θ represents the cosine of the angle between the imaging plane and the surface normal.

To determine cos θ from the 3D surface of a curved phantom, a surface mesh was created in MATLAB of the known geometry of the curved phantom, and the surface normal was calculated in terms of X, Y, and Z at each mesh point. The cosine angle (angle correction factor) between the surface normal and the imaging direction was calculated and applied to DR signals collected from curved phantoms. In uniform light source illumination, the angle between the illumination direction and surface normal (β) is the same as θ. In Gaussian light source illumination, this β angle differs from θ angle, and applying a correction model based on both the angles (β and θ) overcorrects the DR intensity signal. This is because the Gaussian light source illuminating the curved tissue surface at an angle tends to escape before it interacts with the tissue. Hence, the angle-based correction model in our case is based on the angle between the detection direction and the surface normal.

#### 2.1.3 Model 3: height and angle correction

In the third model, both the height correction from model 1 and the angle correction from model 2 are simultaneously implemented. When a surface is inclined, both its height and the angle of inclination influence the detected DR signal. Hence, to accurately account for these variations, both height and angle corrections are applied. If the height correction factor is ε and the angle correction factor is cos θ, the height and angle corrected DR signal is given by

$$I_{HA\_\mathrm{Corr}}(x,y)=\frac{I_{\text{raw}}(x,y,\Delta z)}{\left\{\varepsilon (\Delta z)*\cos\;\theta \right\}}.$$ (3)

#### 2.1.4 Model 4: empirical correction

**Fig. 4:**
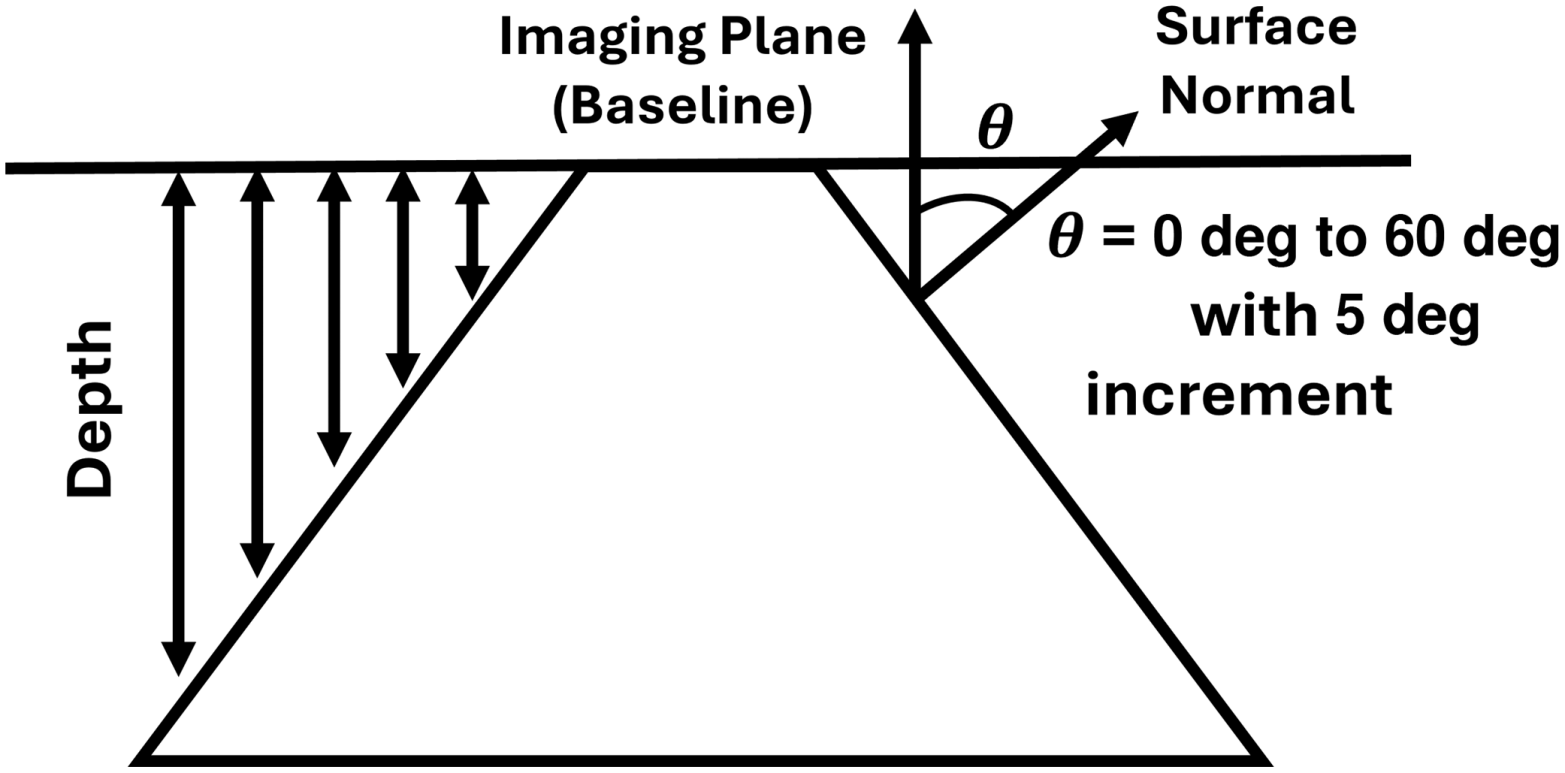
Schematic illustration of the empirical correction factor calculation method. The diagram shows an imaging geometry where the imaging plane (baseline) is placed on the top plane of the object surface. The surface normal is oriented vertically, and the angle of signal collection, θ, is defined as the angle between the surface normal and the imaging direction. θ varies from 0 to 60 deg in 5 deg increments. At each angle θ and depth, the 2D correction factor was calculated as a function of angle and depth.

The Lambertian angle correction is dependent on the angle between the surface normal and the camera axis. However, the Lambertian angle correction is effective for surface inclination angles below 40 deg.^12^ In addition, the Lambertian correction tends to overcorrect the DR signal at a higher surface normal angle.^14^ The possible reason could be that when the angle has a higher inclination, the angle is so high that the signal cannot reach the detector. As a result, when the correction factor is applied, the signal could possibly get overcorrected. Hence, an empirical correction was also developed to account for height and angle together (see Fig. 4).

To develop empirical correction, a rhombus-shaped three-layer tissue phantom model has been created with a fixed angle from 0 to 60 deg with 5 deg increment. The correction factors have been calculated by taking the ratio of the DR signal from each rhombus-shaped phantom to the flat phantom signal. For each specific angle, the depth of each surface point location was calculated from 0 to 1 cm with 0.1 cm increment. The empirical correction factor was calculated for each angle and depth range. If the empirical correction factor is given by ρ at each pixel location for a specific angle and depth, the corrected signal $I_{E\_\mathrm{corr}}$ is

$$I_{E\_\mathrm{corr}}(x,y)=\frac{I_{\text{raw}}(x,y,\Delta z)}{\rho (\Delta z)}.$$ (4)

### 2.2 Study I: Effect of Correction Models on Diffuse Reflected Signals on Simulated Curved Phantoms

**Fig. 5:**
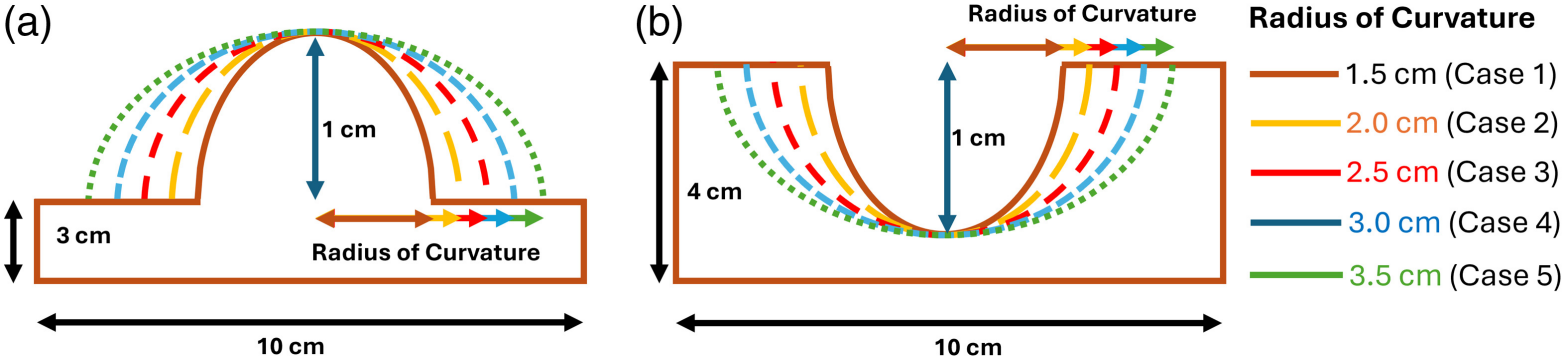
Convex geometry model (a) and concave geometry model (b) with different radii of curvature.

The developed correction models were applied to the three-layered simulated curved phantoms. As the foot surface and the wound surface are curved, the curved surface can have concave and convex geometry. Hence, initially two types of curved geometries have been created in MCMatlab, i.e., concave and convex geometries with different radii of curvature (1.5 to 3.5 cm), as shown in Fig. 5.

**Fig. 6:**
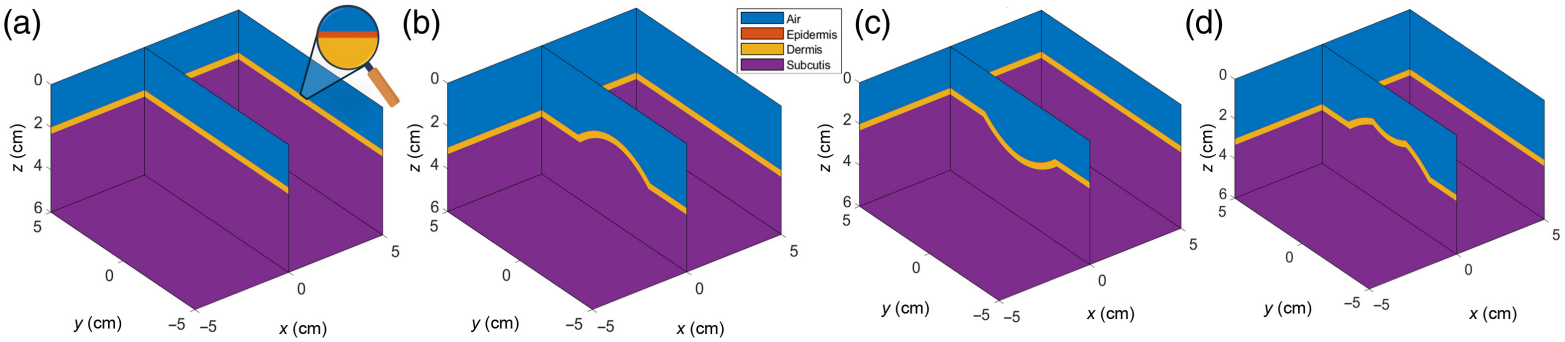
Simulation models of (a) flat surface phantom, (b) convex curved phantom, (c) concave curved phantom, and (d) irregular wound-mimicking phantom in MCMatlab.

In addition, an irregular wound-mimicking phantom (containing a convex and a concave surface) was simulated for validation studies. Figure 6 shows the simulation models of the three-layered flat phantom, convex and concave curved phantoms, and an irregular wound-mimicking phantom.

MC simulations were carried out via MCMatlab across all the simulated phantoms using a Gaussian light source (at 690 and 830 nm), and the DR signal was obtained from the top epidermal surface of each phantom.

**Fig. 7:**
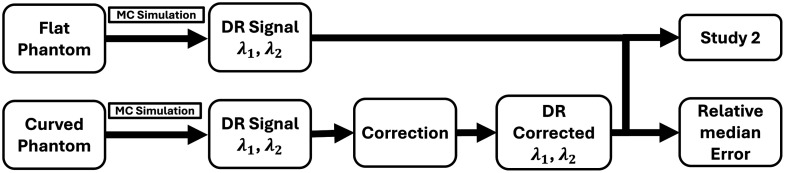
Flowchart of curvature correction models (1 to 4) applied to the DR signals obtained from the curved phantoms and compared with those obtained from the flat phantom surface.

All the correction models (1 to 4) were applied to the DR signal obtained from the various curved phantom surfaces at each illumination wavelength. This DR signal was compared with the DR signal obtained from the flat phantom’s surface (as shown in Fig. 7). The effect of each correction model was determined across all the curved phantom geometries via median relative error calculations to determine the best model that minimizes the error in DR signals due to phantom curvature.

### 2.3 Study II: Effect of Correction on Hemoglobin Parameters

**Fig. 8:**
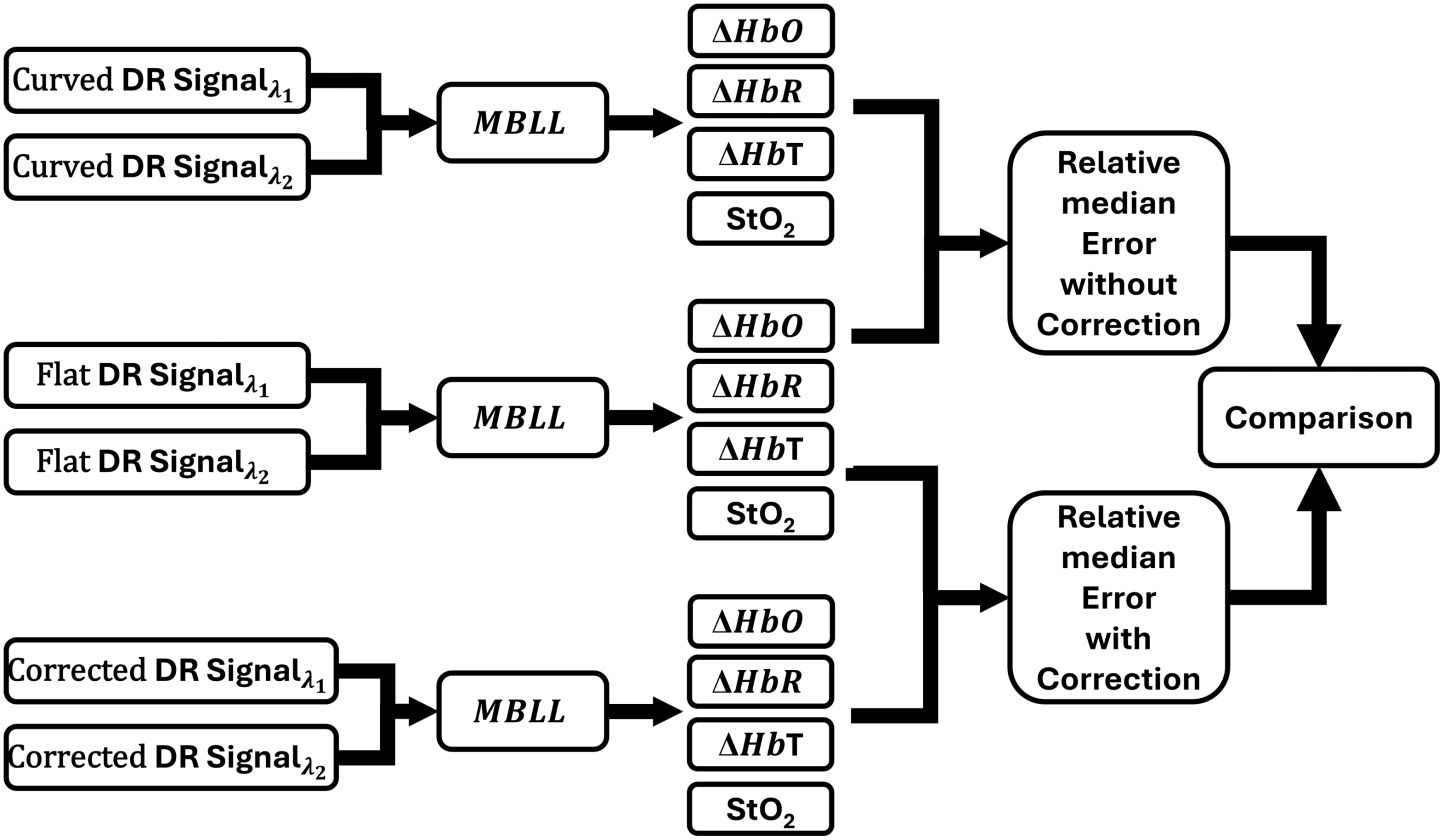
Flowchart to illustrate the effect of curvature correction on hemoglobin parameters—oxyhemoglobin (ΔHbO), deoxyhemoglobin (ΔHbR), total hemoglobin (ΔHbT), and tissue oxygen saturation (${\mathrm{StO}}_{2}$)—estimated from DR signals at two wavelengths ($\lambda_{1}$—690 nm and $\lambda_{2}$—830 nm). Three data sets were analyzed: reference DR signals from a flat phantom, uncorrected DR signals from curved geometries, and curvature-corrected DR signals. The MBLL was applied to each set to extract the hemoglobin parameters. Relative errors were calculated by comparing the curved (without correction) and corrected signal (with correction) against the flat surface signal. The comparison assessed the effectiveness of the correction models in reducing errors in physiological parameters caused by phantom curvature.

The effect of the curvature correction models on hemoglobin parameters was also determined. Modified Beer–Lambert’s Law (MBLL) was applied to the simulated DR signals obtained from both the NIR wavelengths to estimate the hemoglobin parameters in terms of oxy-, deoxy-, total hemoglobin, and oxygen saturation. A simulated reference DR signal (at both wavelengths) obtained from a white diffusing sheet (see Table 1 for the optical properties) was used during MBLL calculations. The details of the MBLL and the equations used to obtain the hemoglobin parameters are described in detail elsewhere.^25^^,^^26^ The uncorrected and the corrected DR signals of the curved phantoms (using the best correction model from study I) were further used to determine the hemoglobin parameters. The effect of curvature correction models was determined across all the curved phantom geometries via median relative error calculations to determine the best model that minimizes the error in hemoglobin parameters due to phantom curvature. The complete flowchart of the study is shown in Fig. 8.

## 3 Results and Discussion

The effect of the curvature correction model(s) on the corrected DR signals (in study I) and hemoglobin parameters (in study II) obtained from different curved phantom geometries is compared, and the relative percentage errors are calculated, as given below.

$$\text{Relative Percentage Error}=\frac{\text{Flat Surface Signal}-\text{Curved Surface Signal}}{\text{Flat Surface Signal}}\times 100\%.$$ (5)

The relative error calculations were performed on the $10\times 10\;{\mathrm{cm}}^{2}$ phantom’s top surface, excluding 0.5 cm of the edges on all sides (to avoid edge effects or, in other words, the boundary effects).

### 3.1 Study I: Effect of Correction Models on Diffuse Reflectance Signals

#### 3.1.1 Concave geometry

**Fig. 9:**
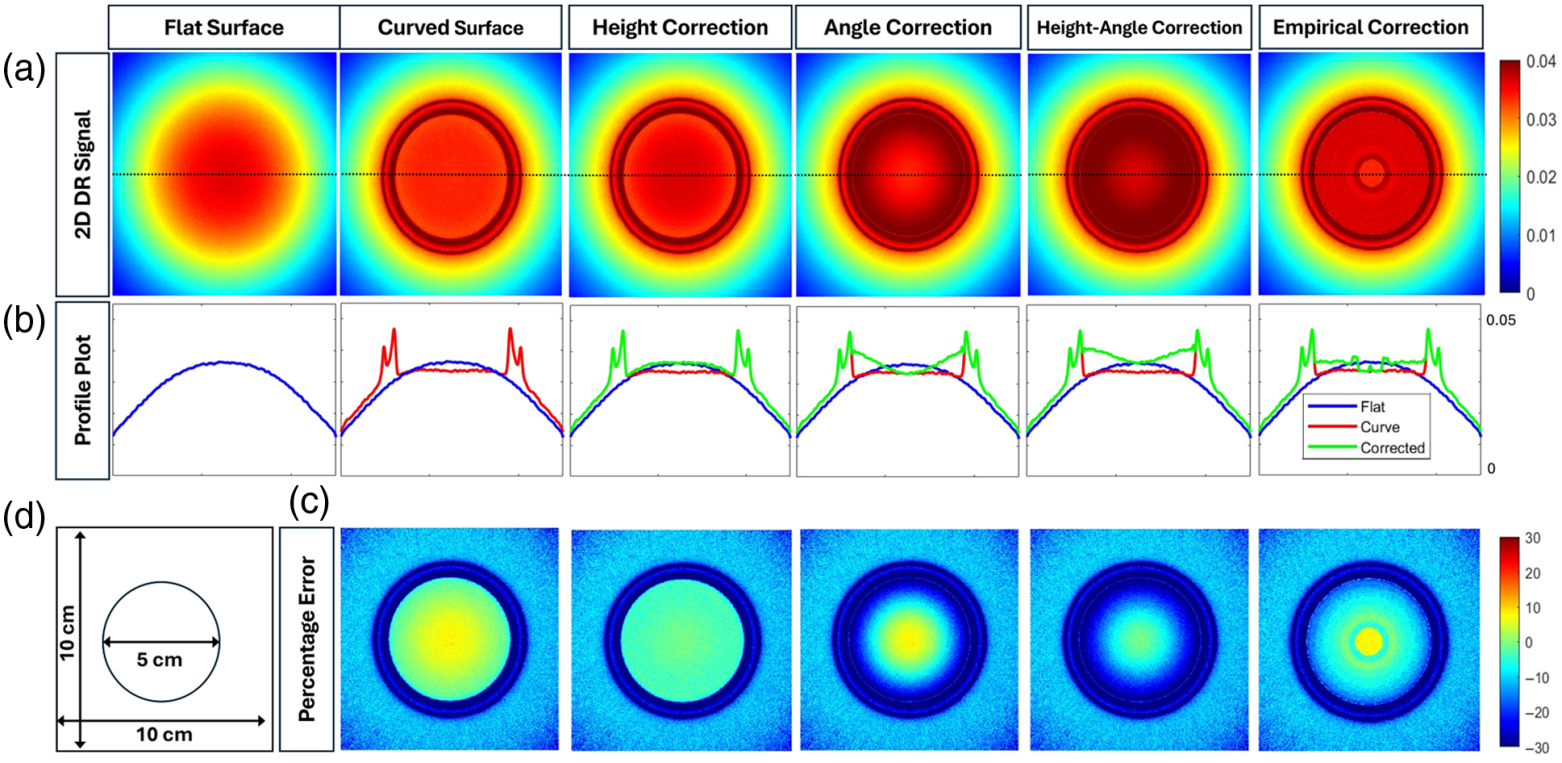
(a) 2D spatial maps of the detected DR signals obtained from a flat phantom geometry, the concave geometry with and without the various correction models applied. (b) The profile plot of each DR signal along the y-axis at x=5 (center location). The blue lines indicate the reference signals, red lines represent the uncorrected signal of the curved surface, and the green lines represent the corrected signal with the corresponding correction model. (c) The relative percentage error obtained from the concave curved surface with respect to the flat surface for before and after correction. (d) The image of a 10  cm×10  cm phantom’s top surface with a 5 cm diameter concave region.

Figure 9(a) shows the pseudo-color maps of the simulated DR signal distributions for a flat phantom and a sample concave (curved) phantom geometry with a radius of curvature of 2.5 cm at 690 nm wavelength. Four different correction models were applied: height correction (model 1), angle correction (model 2), combined height/angle correction (model 3), and empirical correction (model 4).

Figure 9(b) shows the x-profile of the DR signal at the midpoint of the y-axis (y=5  cm). Figure 9(c) shows the relative error in 2D DR signals between the surface of the flat phantom and the uncorrected or corrected curved phantom.

The 1D profile plots and the relative error maps demonstrate that the correction models tend to overcorrect the signal in concave curved geometries. The height correction improves the signal close to the flat surface. However, the angle correction model alone introduces overcorrection at the interface of curved regions and flat regions. Both the combined height/angle correction and empirical correction models also overcorrect the signal. In other words, concave curved phantoms do not need correction, possibly because the DR signals emerging from the concave surface converge and enhance the detected DR. This was confirmed when comparing the simulated DR signals from the center of the concave curved regions with respect to that from the flat regions of simulated concave curved geometries during MC simulation studies using both uniform and Gaussian light sources (plots not shown here for brevity). The DR signals from the concave surface were similar to those obtained from the flat surfaces, confirming that light converged within the concave curved surface. Hence, any curvature correction model applied to concave curved regions was overcorrecting the DR signals. This conclusion is further confirmed by the median of relative errors given in Table 3, where correction models have the same or increased errors when implementing correction models at both the 690 and 830 nm wavelengths.

**Table 3:** Median of the relative error (as percentages) in the DR signals without and with implementation of each correction model in concave curved phantoms at both 690 and 830 nm Gaussian light source illumination.

Wavelength	690 nm					830 nm				
Radius of curvature (cm)	Without correction	With correction				Without correction	With correction			
		Model 1	Model 2	Model 3	Model 4		Model 1	Model 2	Model 3	Model 4
1.5	−11.80	−11.76	−12.38	−12.42	−12.23	−13.36	−13.30	−13.94	−13.96	−13.84
2.0	−12.26	−12.23	−13.25	−13.46	−12.83	−14.07	−14.01	−15.07	−15.26	−14.77
2.5	−11.67	−11.63	−13.20	−13.70	−12.39	−13.52	−13.46	−15.03	−15.51	−14.41
3.0	−10.36	−10.33	−12.63	−13.52	−11.55	12.18	−12.13	−14.48	−15.35	−13.58
3.5	−8.15	−8.35	−12.22	−13.65	−10.72	9.88	−10.08	−13.94	−15.41	−12.82

#### 3.1.2 Convex geometry

**Fig. 10:**
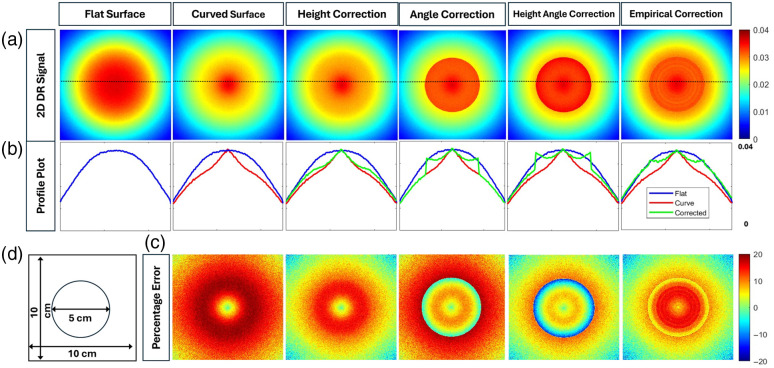
(a) 2D spatial maps of the detected DR signals obtained from a flat phantom geometry and a convex geometry, with and without the application of various correction models. (b) The profile plots of each DR signal along the x-axis at y=5 (dotted line center location). The blue lines indicate the reference signals from the flat surface, the red lines represent the uncorrected signal from the convex curved surface, and the green lines represent the corrected signals corresponding to each correction model. (c) The relative percentage error obtained from the convex curved surface with respect to the flat surface, both before and after applying the correction models. (d) The image of a 10  cm×10  cm phantom’s top surface with a 5 cm diameter convex region.

Figure 10 shows the pseudo-color maps of the simulated DR signal distributions for convex (curved) phantom geometry with a radius of curvature of 2.50 cm at 690 nm wavelength, along with the 1D x-profile plots and relative error plots (similar to those in the concave phantom geometry results).

The DR signals from the convex region of the curved geometry are notably reduced compared with those from a flat surface. This is possibly from the divergence of the DR signal from a convex surface that reduces the overall intensity of the detected DR signal, unlike a convergent DR signal of increased intensity from concave surfaces (as described in Sec. 3.1.1). The height correction model provided minimal improvement, indicating that height correction alone is insufficient to recover signal accuracy in convex geometries. The angle correction model, on the other hand, overcorrected the signal. Typically, the Lambertian angle correction is effective for surface inclination angles below 40 deg.^12^ When these angles between the surface normal and the camera axis are greater than 40 deg, as observed in our convex geometries, the Lambertian angle correction overcorrects the DR signal. The combined height and angle correction model does reduce the overall error due to curvature, whereas the empirical correction model seems to be the closest to matching the DR signals from a flat surface. These observations were further confirmed by the median of relative error given in Table 4, where the empirical correction model yielded the least median relative error when correcting the curvature of the convex geometries at all radii of curvatures and at both the 690 and 830 nm wavelengths.

**Table 4:** Median of the relative error (as percentages) in the DR signals without and with implementation of each correction model in convex curved phantoms at both 690 and 830 nm with Gaussian light source illumination.

Wavelength	690 nm					830 nm				
Radius of curvature (cm)	Without correction	With correction				Without correction	With correction			
		Model 1	Model 2	Model 3	Model 4		Model 1	Model 2	Model 3	Model 4
1.5	13.22	5.40	12.40	4.45	−3.48	13.85	5.68	12.99	4.66	3.73
2.0	13.26	5.59	11.68	4.04	−3.24	13.82	5.82	12.18	4.17	−3.55
2.5	13.60	6.20	10.87	4.10	−2.40	14.16	6.46	11.27	4.24	−2.76
3.0	13.98	6.92	10.57	4.50	−1.52	14.49	7.14	10.93	4.66	−1.82
3.5	14.21	7.76	10.54	4.88	−0.15	14.68	7.94	10.90	5.00	−0.41

#### 3.1.3 Wound-mimicking geometry

**Fig. 11:**
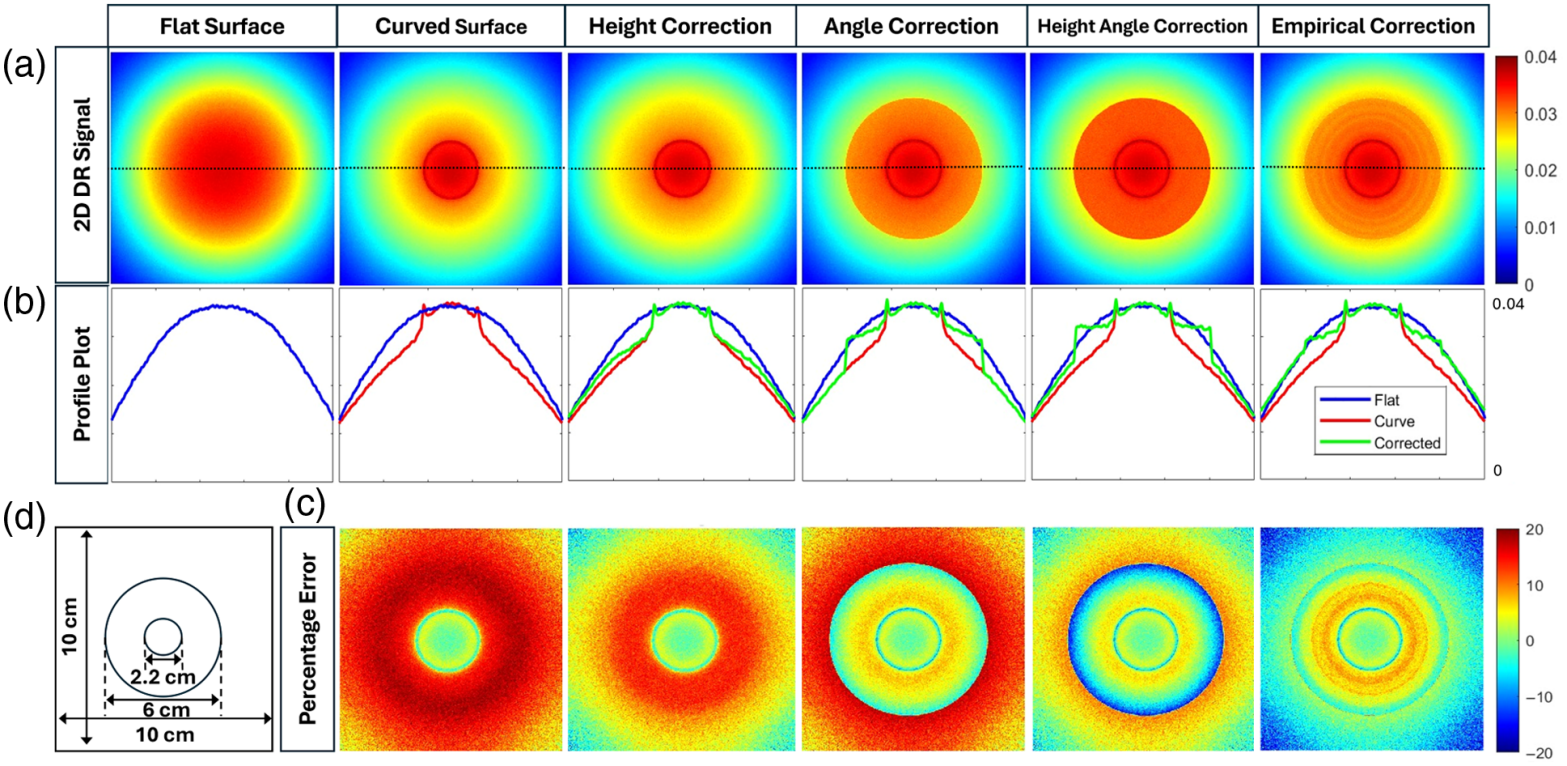
(a) 2D spatial maps of the detected DR signals obtained from a flat phantom geometry and a wound-mimicking phantom surface, with and without the application of various correction models. (b) The profile plots of each DR signal along the y-axis at x=5 (center location). The blue lines indicate the reference signals from the flat surface, the red lines represent the uncorrected signal from the wound-mimicking surface, and the green lines represent the corrected signals corresponding to each correction model. (c) The relative percentage error obtained from the wound-mimicking surface with respect to the flat surface, both before and after applying the correction models. (d) The image of a 10  cm×10  cm phantom’s top surface with a central concave region (2.2 cm diameter) surrounded by a convex (6 cm diameter) region.

Figure 11 shows the pseudo-color 2D DR plot, 1D x-profile plot, and relative error plots obtained from the wound-mimicking phantom surface when illuminated with a 690 nm Gaussian light source. As the correction models did not have any improvement when applied to concave surfaces (as described in Sec. 3.1.1), all the correction models were applied to only the convex surfaces of the wound-mimicking phantom, and not to the concave surfaces.

The height correction and angle corrections could not individually reduce the error, but when both were used together, the error reduced. However, the empirical correction provided the least error, which indicates the effectiveness of the previous results for the convex curved phantom. These observations were further confirmed from the relative median error for both 690 and 830 nm wavelengths in Table 5. Hence, the empirical corrected signal was used to calculate the hemoglobin parameters in study II.

**Table 5:** Median of the relative error (as percentages) in the DR signals without and with implementation of each correction model in wound-mimicking phantoms at both 690 and 830 nm Gaussian light source illumination.

Wavelength (nm)	Gaussian light source				
	Without correction	With correction			
		Model 1	Model 2	Model 3	Model 4
690	13.81	6.57	9.32	3.16	−1.81
830	14.32	6.83	9.6	3.13	−2.18

In summary, the empirical correction model (model 4) reduced the effect of curvature on DR signals in both the convex and wound-mimicking phantoms. None of the curvature correction models impacted the DR signals obtained from concave phantoms. Hence, study II will only focus on convex and wound-mimicking phantoms when assessing the effect of correction models on hemoglobin parameters.

### 3.2 Study II: Effect of Correction Models on Hemoglobin Parameters

The DR signals of the curved phantoms (both uncorrected and corrected signals) obtained at 690 and 830 nm from the convex and wound-mimicking surfaces were, in turn, used along with the MBLL model to estimate the hemoglobin parameters in terms of ΔHbO, ΔHbR, ΔHbT, and ${\mathrm{StO}}_{2}$. The hemoglobin parameters of the three-layered skin phantom of a convex curved phantom (with varying radii of curvature) and a wound-mimicking geometry were calculated. Only the DR signals corrected using the empirical correction model were employed to assess their impact on reducing errors in hemoglobin parameters because they provided the least error in both the convex and wound-mimicking geometries. As there is no effect of curvature on concave geometries, the hemoglobin parameter has not been calculated for concave geometries.

#### 3.2.1 Convex geometry

**Fig. 12:**
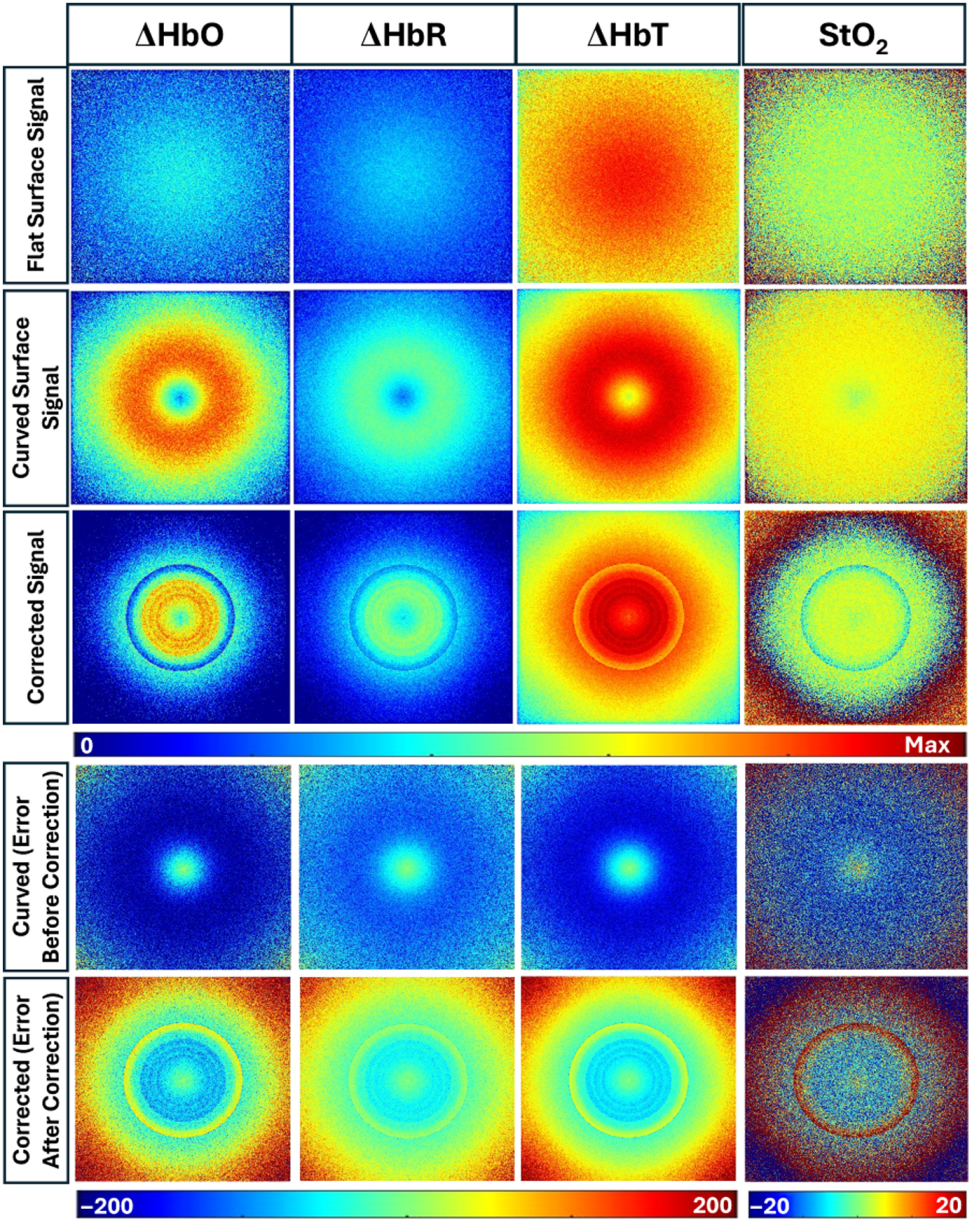
Hemoglobin parameter maps (ΔHbO, ΔHbR, ΔHbT, and ${\mathrm{StO}}_{2}$) and corresponding relative error maps for a flat surface, an uncorrected convex curved phantom, and a corrected curved phantom surface. The convex surface has a radius of curvature of 2.5 cm. In the color bars, blue and red denote low- and high-intensity values of the hemoglobin parameters, respectively. The relative error maps illustrate the effect of the curvature of each parameter from the flat surface. In the relative error maps, blue represents minimal error, whereas red indicates higher error, emphasizing the impact of tissue curvature and the effectiveness of the applied empirical correction model.

Figure 12 shows the pseudo-color distributions of ΔHbO, ΔHbR, ΔHbT, and ${\mathrm{StO}}_{2}$ for both the flat phantom and curved phantom without and with empirical correction. The empirical corrected signal has been chosen because it provides the least error for the DR signal.

The hemoglobin parameter maps from the flat phantom exhibit spatially uniform distributions across all four parameters. In contrast, the convex curved phantom demonstrates distinct variation in all the hemoglobin parameter calculations. The curved geometry showed an increased ΔHbO than that obtained from a flat phantom. The DR signal from a convex geometry surface is reduced when compared with that obtained from a flat surface (as given in Fig. 10). This reduction in detected DR signal translates to an increased absorption, which further implies that the hemoglobin concentration in terms of ΔHbO is higher (as shown in Fig. 12). A similar trend was observed in ΔHbR and ΔHbT maps. On the contrary, the effect of curvature appeared smaller in ${\mathrm{StO}}_{2}$ maps compared with the other hemoglobin parameter maps. Upon applying the correction model, the error further reduced, further improving the accuracy of our empirical correction model for oxygen saturation maps.

The median relative errors across all the hemoglobin parameters with and without applying curvature corrections to the DR signals at 690 and 830 nm are shown in Table 6.

**Table 6:** Median of the relative error (as percentages) of hemoglobin parameters without correction and with empirical correction model in convex curved phantoms for Gaussian light source illumination.

Hemoglobin parameters	1.5 cm		2.0 cm		2.5 cm		3.0 cm		3.5 cm	
	Before	After	Before	After	Before	After	Before	After	Before	After
ΔHbO	−174.73	39.28	−173.29	36.13	−172.77	28.62	−172.51	18.25	−171.76	−0.70
ΔHbR	−97.91	22.01	−98.20	19.83	−99.18	14.41	−100.24	8.38	−101.08	−2.44
ΔHbT	−141.05	35.24	−140.90	33.08	−142.04	25.27	−143.58	16.78	−144.95	4.34
${\mathrm{StO}}_{2}$	−14.23	3.04	−13.79	2.58	−13.45	1.00	−13.10	−0.57	−12.70	−1.91

#### 3.2.2 Wound-mimicking geometry

**Fig. 13:**
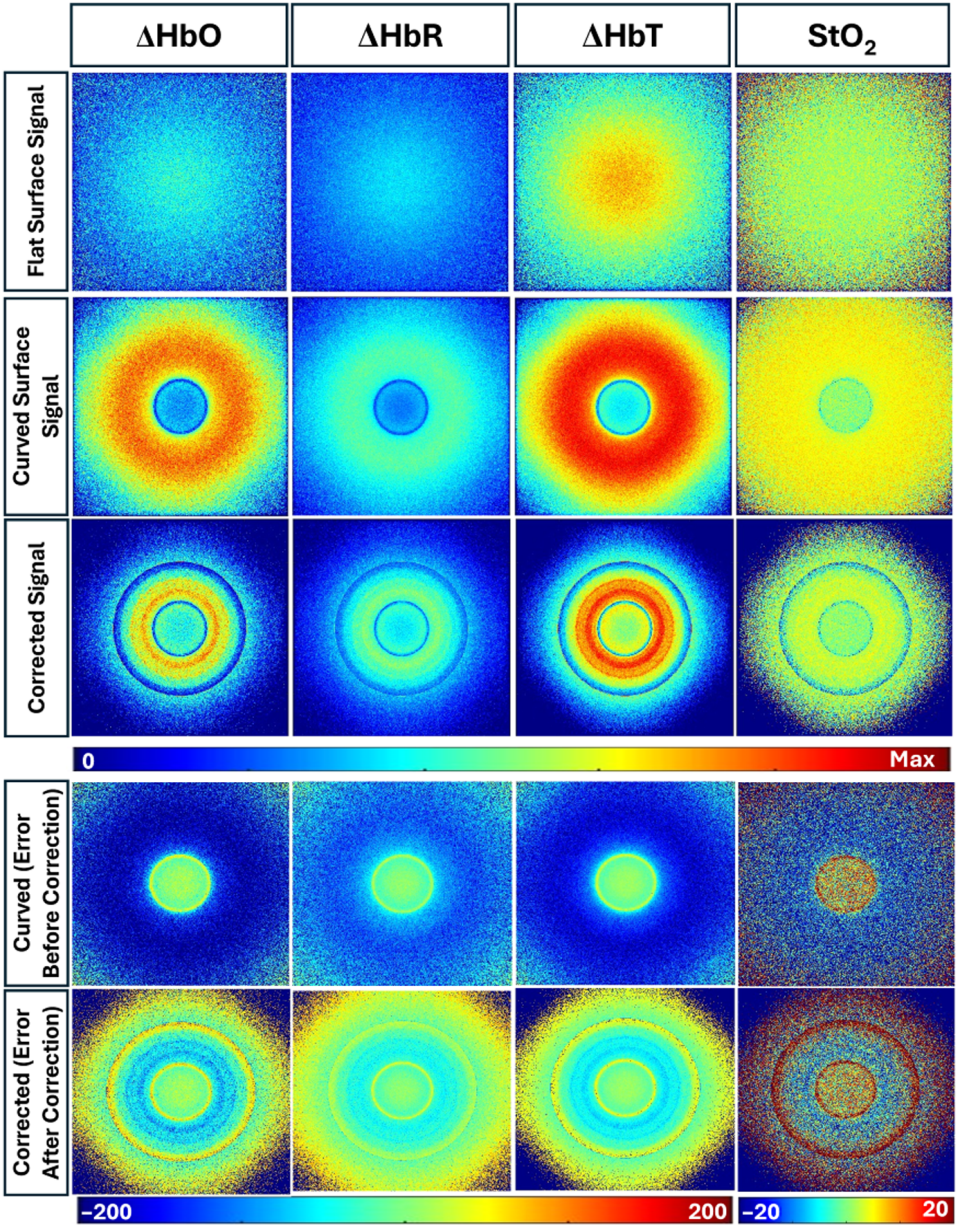
Hemoglobin parameter maps (ΔHbO, ΔHbR, ΔHbT, and ${\mathrm{StO}}_{2}$) and corresponding relative error maps for a flat surface, an uncorrected, and a corrected wound-mimicking surface using Gaussian illumination. In the color bars, blue and red denote low- and high-intensity values of hemoglobin parameters, respectively. The relative error maps before and after correction illustrate the effect of the curvature of each condition from the flat surface. In the relative error maps, blue represents minimal error, whereas red indicates higher error, emphasizing the impact of tissue curvature and the effectiveness of the applied empirical correction model.

Figure 13 presents the pseudo-color distributions of hemoglobin parameters—ΔHbO, ΔHbR, ΔHbT, and ${\mathrm{StO}}_{2}$—for an irregular wound-mimicking phantom, which includes both concave and convex curvatures illuminated with a Gaussian light source. Similar to the convex geometry case in Fig. 12, the ${\mathrm{StO}}_{2}$ map showed a relatively homogeneous distribution, consistent with a flat phantom signal. However, the normalized maps of ΔHbO, ΔHbR, and ΔHbT show clear spatial variation compared with a flat surface within the convex regions caused by curvature.

After applying the empirical correction model, the 2D hemoglobin parameter distribution maps improved in the convex curved regions across all hemoglobin parameters, resulting in a reduction in relative error. Although the correction improved the corrected 2D hemoglobin parameter distribution, the corrected distributions remained less homogeneous compared with the flat surface maps (similar to that in the convex geometry case in Fig. 12). However, the overall median relative error significantly reduced across all the hemoglobin parameter maps, as given in Table 7.

**Table 7:** Median of the relative error (as percentages) of hemoglobin parameters without correction and with empirical correction model in wound-mimicking phantoms for Gaussian light source illumination.

Correction options	Gaussian light source			
	ΔHbO (%)	ΔHbR (%)	ΔHbT (%)	${\mathrm{StO}}_{2}$ (%)
Without correction	−171.71	−98.82	−141.83	−12.48
With correction	8.76	7.08	6.89	2.3

In summary, curvature correction is essential, and the empirical correction model reduced the errors in the hemoglobin parameter maps, especially the ${\mathrm{StO}}_{2}$ maps. Future work will involve extensive phantom studies on realistic foot models to validate the curvature correction models, prior to implementing them during *in vivo* imaging of wounds of varying curvatures. When validating on realistic and anatomically complex phantoms, we will obtain the 3D depth maps of these complex geometries using a stereoscopic camera. Preliminary studies were performed using a miniature stereoscopic camera to obtain the 3D depth maps of the imaged curved surfaces. Our preliminary results using the stereoscopic camera could obtain the surface depths with <1% error and a resolution of 2 mm.^27^ The effect of the measurement errors in surface geometry and how much they will impact the tissue oxygenation estimates will also be a part of the future phantom studies, where the stereoscopic camera will be implemented to obtain the 3D surface depth maps.

## 4 Conclusion

In this work, we have developed and validated mathematical models to correct the tissue curvature effect in DR signals obtained during NIRS imaging. Using Monte Carlo simulations for light propagation in MCMatlab, four correction models—height correction, angle correction, combined height/angle correction, and an empirical correction—were developed. The correction models were applied and verified on three-layered concave and convex curved skin phantoms with different radii of curvature from 1.5 to 3.5 cm using Gaussian illumination (at 690 and 830 nm). The analysis shows the effectiveness of each correction model on the phantoms of different shapes. The results show that concave surfaces inherently converge DR signals, yielding no effect of curvature correction for concave geometries. On the other hand, convex geometries introduce divergence in the DR signal, requiring curvature correction. The median error shows that the empirical correction model reduced the error in the range of 13%–14% to 0.5%–3.75% and provided the least error after correction. In addition, the applicability of the correction models was applied and verified on a wound-mimicking phantom with complex geometry, which also indicates that the empirical correction model brought the error from 13%–14% to 1%–2% after correction. Furthermore, hemoglobin parameters were calculated using the MBLL without correction and with correction of the DR signal of the convex curved phantom and wound-mimicking phantom. It was observed that the empirical correction model significantly reduces the error for hemoglobin parameter calculation after correction, especially in ${\mathrm{StO}}_{2}$ estimations (to <2.5% median relative error).

Future work involves validating the correction model experimentally on curved phantoms and incorporating the curvature correction model in our in-house smartphone-based NIRS imaging device (or SPOT). As the NIRS device is handheld and portable with potential as a bedside imaging tool for DFUs, the depths between the imaging plane and the tissue curvature cannot be determined using benchtop profilometers, as used by researchers in the past. Hence, our ongoing work involves the use of a miniature stereoscopic camera to obtain the 3D surface topology to determine the imaging depths required for height correction in curved tissues.^26^^,^^27^

## Data Availability

All the data are simulated via Monte Carlo simulations using MCMatlab. The code and data are available upon request made to the corresponding author.
